# Zinc Pharmacotherapy for Elderly Osteoporotic Patients with Zinc Deficiency in a Clinical Setting

**DOI:** 10.3390/nu13061814

**Published:** 2021-05-27

**Authors:** Masaki Nakano, Yukio Nakamura, Akiko Miyazaki, Jun Takahashi

**Affiliations:** Department of Orthopaedic Surgery, School of Medicine, Shinshu University, 3-1-1 Asahi, Matsumoto, Nagano 390-8621, Japan; masakin04@shinshu-u.ac.jp (M.N.); akmmsi73@gmail.com (A.M.); jtaka@shinshu-u.ac.jp (J.T.)

**Keywords:** bone mineral density, elderly patients, osteoporosis, zinc deficiency, zinc pharmacotherapy

## Abstract

Although there have been reported associations between zinc and bone mineral density (BMD), no reports exist on the effect of zinc treatment in osteoporotic patients. Therefore, we investigated the efficacy and safety of zinc pharmacotherapy in Japanese elderly patients. The present investigation included 122 osteoporotic patients with zinc deficiency, aged ≥65 years, who completed 12 months of follow-up. In addition to standard therapy for osteoporosis in a clinical setting, the subjects received oral administration of 25 mg zinc (NOBELZIN^®^, an only approved drug for zinc deficiency in Japan) twice a day. BMD and laboratory data including bone turnover markers were collected at 0 (baseline), 6, and 12 months of zinc treatment. Neither serious adverse effects nor incident fractures were seen during the observation period. Serum zinc levels were successfully elevated by zinc administration. BMD increased significantly from baseline at 6 and 12 months of zinc treatment. Percentage changes of serum zinc showed significantly positive associations with those of BMD. Bone formation markers rose markedly from the baseline values, whereas bone resorption markers displayed moderate or no characteristic changes. Additive zinc supplementation may contribute to BMD augmentation ensuing the prevention of fracture occurrence in elderly osteoporotic patients with zinc deficiency.

## 1. Introduction

Zinc is an essential nutritional trace element which acts as an important co-factor for a number of enzymes including DNA and RNA polymerases, and is necessary for the organisms [1,2]. In general, the human body contains 1.5–3 g of zinc and approximately 0.1% of the amount is excreted daily, and thus needs to be supplemented through dietary intake [3]. Retardation in bone growth, as well as dermatitis, anemia, and immune system dysfunction, is reportedly associated with zinc deficiency [4,5]. Moreover, a large proportion of zinc body burden is known to be located within the skeleton [6]. Zinc in the bone has been shown to be accumulated in the osteoid layer prior to mineralization [7].

Several osteoporosis-related risk factors, such as aging, menopause, or unloading, are demonstrated to correlate with decreased bone zinc amounts [8]. Since women with osteoporosis excrete significantly higher zinc, above 0.8 mg zinc/g creatinine, in urine, urinary zinc has been considered to be a possible bone resorption marker in postmenopausal women [6,9]. In addition, significant associations of low serum levels of zinc as well as copper, iron, and magnesium with diminished bone mineral density (BMD) and the risk of osteoporosis have been reported in postmenopausal women [10,11,12]. Therefore, the importance of zinc supplementation for bone health maintenance has emerged.

Zinc has been suggested to possess a beneficial effect on the fracture healing in an animal model and in patients with traumatic bone fracture [13,14]. Moreover, the efficacy of oral zinc administration for the protection of bone resorption caused by unloading, low-calcium and -vitamin D diet, and estrogen deficiency, as well as diabetes or steroid induced osteoporosis, was also demonstrated in rats [8]. However, no reports exist on the effect of zinc treatment in patients with osteoporosis. Therefore, we investigated the efficacy and safety of oral administration of zinc acetate dihydrate formulation (NOBELZIN^®^, an only approved drug for zinc deficiency in Japan) in Japanese elderly osteoporotic patients with zinc deficiency in combination with a standard practice for osteoporosis.

## 2. Materials and Methods

This study was approved by the Institutional Review Board of Shinshu University Hospital, Japan, prior to the commencement (approval number 4412). The research procedure was conducted in accordance with the ethical guidelines of the 2013 Declaration of Helsinki. A written informed consent was obtained from all participants enrolled in this study.

### 2.1. Study Subjects

The inclusion criteria of this study were primary and secondary osteoporotic patients, aged ≥65 years, with zinc deficiency (serum zinc <60 µg/dL) or marginal zinc deficiency (serum zinc 60–80 µg/dL) [15] who had consecutively received more than 1 year of osteoporosis treatment prior to the registration. Subjects who discontinued zinc pharmacotherapy within 1 year were excluded from data analysis. Osteoporosis was diagnosed according to the revised criteria established by the Japanese Society for Bone and Mineral Research [16]. A total of 131 male and female patients were prospectively recruited from our institutions between July 2019 and February 2020. In addition to the standard medication for osteoporosis in a clinical setting, the subjects received oral administration of 25 mg zinc (83.92 mg zinc acetate dihydrate) (NOBELZIN^®^, Nobelpharma, Tokyo, Japan) twice a day. After exclusion of 9 participants who experienced nausea and discontinued the zinc treatment, the analysis of this investigation included 122 patients (male, 23; female, 99) who completed 12 months of follow-up.

### 2.2. Data Collection

Serum levels of albumin, hemoglobin A_1c_, zinc, copper, iron, magnesium, calcium, phosphorus, 25(OH)D, and 1,25(OH)_2_D_3_ were assayed at SRL, Inc. (Tokyo, Japan). Zinc and copper were measured by the colorimetric method, while iron and magnesium were determined by the nitroso-PSAP method and xylidyl blue method, respectively. The concentration of 25(OH)D was determined by chemiluminescent enzyme immunoassay (CLEIA) and that of 1,25(OH)_2_D_3_ was measured by radioimmunoassay. BMD at the lumbar spine (L1–4), bilateral total hips, and femoral necks was measured using a dual-energy X-ray absorptiometry (DXA; PRODIGY, GE Healthcare, Chicago, IL). Moreover, as bone turnover markers, serum procollagen type 1 N-terminal propeptide (P1NP), serum bone-specific alkaline phosphatase (BAP), urinary cross-linked N-terminal telopeptide of type 1 collagen (NTx), and serum tartrate-resistant acid phosphatase-5b (TRACP-5b) were determined by SRL, Inc. BAP and NTx were assayed by CLEIA, while P1NP and TRACP-5b were measured by electrochemiluminescence immunoassay and enzyme immunoassay, respectively. BMD data were collected at −6 (i.e., 6 months before the initiation of zinc administration), 0 (baseline), 6, and 12 months of zinc pharmacotherapy, and laboratory parameters were determined at 0, 6, and 12 months.

### 2.3. Statistical Analysis

The baseline characteristic data of study subjects are reported as the mean ± standard deviation (SD), together with the minimum, median, and maximum values. Percentage changes from baseline in BMD and laboratory parameter values were calculated. The significance of differences between the baseline and percentage changes at −6 (for BMD only), 6, or 12 months was evaluated by the Wilcoxon signed-rank testing, while that between genders at each time point was assessed by the Wilcoxon rank-sum test. Pearson’s product-moment correlation and Spearman’s rank correlation were employed to examine the relationships between percentage changes of serum zinc and BMD at 12 months. In addition, we performed the multiple regression analysis for the percentage changes of BMD at 12 months by serum zinc percentage change to validate the associations between circulating zinc and BMD changes. The regression models were adjusted for patient age, body mass index, and serum albumin and hemoglobin A_1c_ levels at registration. All statistical tests were performed using R version 3.6.0 software (https://www.r-project.org/, accessed on 2 July 2019) [17], and a two-tailed *P*-value of <0.05 was considered significantly different.

## 3. Results

### 3.1. Patient Characteristics

The average ± SD age of the 122 osteoporotic male and female patients at registration was 74.5 ± 7.1 years. All subjects were zinc deficient or marginal zinc deficient with serum zinc levels of ≤80 µg/dL, and the median was 63 µg/dL. Seventy-five patients were affected with primary osteoporosis and the rest (*n* = 47) were glucocorticoid-induced osteoporosis (GIO), of which 33 were rheumatoid arthritis (RA) patients. All patients had received a 1 year or more (4.7 ± 3.3 years) treatment for osteoporosis that included bisphosphonates (*n* = 61), denosumab (*n* = 33), romosozumab (*n* = 14), teriparatide (*n* = 9), and selective estrogen receptor modulators (*n* = 5). In addition to oral zinc administration, the subjects were medicated with bisphosphonates (*n* = 42), denosumab (*n* = 15), romosozumab (*n* = 56), and teriparatide (*n* = 9). The baseline characteristics of study patients are summarized in Table 1.

### 3.2. Laboratory Data and BMD Changes

**Figure 1:**
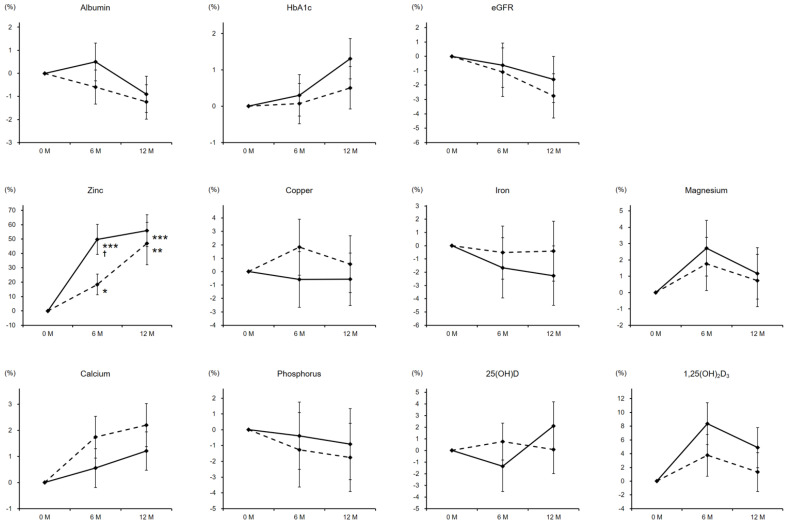
Percentage changes in laboratory parameters during the zinc treatment. Dashed and solid lines indicate the data of male and female patients, respectively. The significance of differences in 6 and 12 months vs. baseline was evaluated by the Wilcoxon signed-rank test (* *P* < 0.05, ** *P* < 0.01, and *** *P* < 0.001), while that between genders at each time point was assessed by the Wilcoxon rank-sum testing (^†^ *P* < 0.05). HbA1c, hemoglobin A_1c_; eGFR, estimated glomerular filtration rate.

Serum zinc levels were successfully elevated by zinc pharmacotherapy in both genders with a relatively higher responsiveness in women. The median values at 6 and 12 months were 89 and 92 µg/dL, respectively. Meanwhile, other laboratory parameters including albumin, hemoglobin A_1c_, estimated glomerular filtration rate, copper, iron, magnesium, calcium, phosphorus, 25(OH)D, and 1,25(OH)_2_D_3_ exhibited no significant changes (Figure 1).

**Figure 2:**
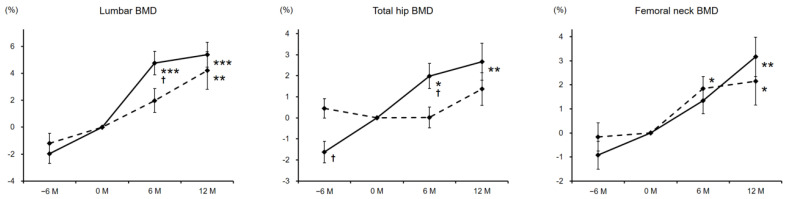
Percentage changes in BMD before and during the zinc treatment. Dashed and solid lines indicate the data of male and female patients, respectively. The significance of differences in −6, 6, and 12 months vs. baseline was evaluated by the Wilcoxon signed-rank test (* *P* < 0.05, ** *P* < 0.01, and *** *P* < 0.001), while that between genders at each time point was assessed by the Wilcoxon rank-sum testing (^†^ *P* < 0.05). BMD, bone mineral density.

BMD at the lumbar spine was significantly increased to 4.2 and 5.4% in male and female patients, respectively, from baseline at 12 months of zinc treatment. Respective percentage changes of total hip BMD at 12 months in male and female patients were 1.4 and 2.7%, and that was statistically significant only in women. Femoral neck BMD also increased significantly at 12 months afterwards (male, 2.1%; female, 3.2%) (Figure 2).

**Figure 3:**
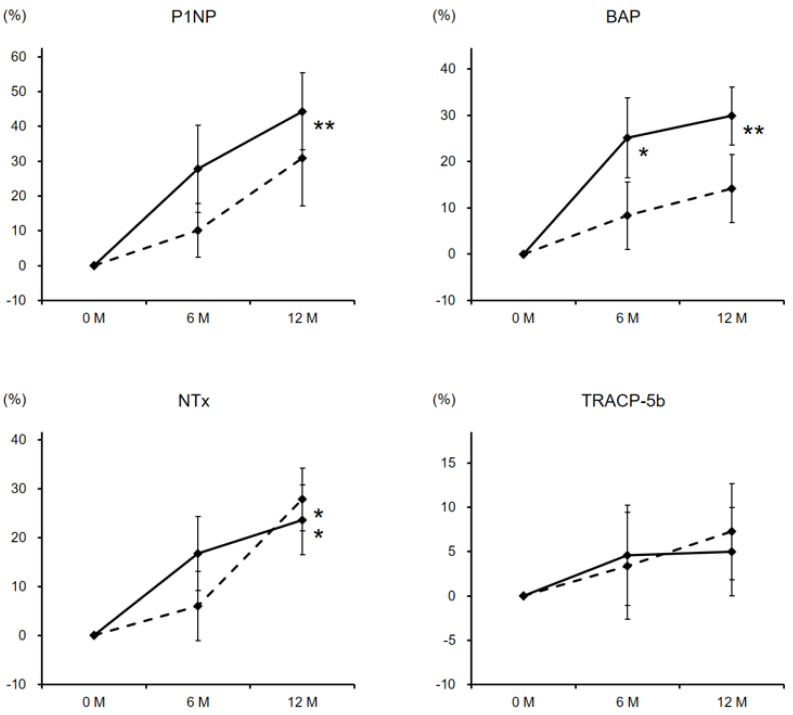
Percentage changes in bone turnover markers during the zinc treatment. Dashed and solid lines indicate the data of male and female patients, respectively. The significance of differences in 6 and 12 months vs. baseline was evaluated by the Wilcoxon signed-rank test (* *P* < 0.05 and ** *P* < 0.01), while that between genders at each time point was assessed by the Wilcoxon rank-sum testing. P1NP, procollagen type 1 N-terminal propeptide; BAP, bone-specific alkaline phosphatase; NTx, cross-linked N-terminal telopeptide of type 1 collagen; TRACP-5b, tartrate-resistant acid phosphatase-5b.

Both bone formation markers, P1NP and BAP, rose markedly from the baseline values at 6 and 12 months of zinc supplementation, especially in women. As for bone resorption markers, though NTx showed a significant elevation at 12 months in both genders, TRACP-5b levels displayed no characteristic changes (Figure 3).

Percentage changes of serum zinc showed significantly positive correlations with those of lumbar (*r* = 0.477, *ρ* = 0.442), total hip (*r* = 0.395, *ρ* = 0.321), and femoral neck BMD (*r* = 0.452, *ρ* = 0.407) (Table 2).

The multiple regression analysis revealed statistically significant associations between percentage changes of serum zinc and BMD. The adjusted odds ratios for lumbar, total hip, and femoral neck BMD were 1.81 (95% confidence interval (CI), 1.39–2.37), 1.77 (95% CI, 1.37–2.23), and 1.86 (95% CI, 1.42–2.42), respectively (Table 3).

### 3.3. Safety Evaluations

Of the 131 subjects enrolled in this investigation, nine patients discontinued zinc administration due to nausea. No other serious adverse effects were seen in physical conditions and/or laboratory data during the observation period, with no incident fractures, as well.

## 4. Discussion

In the present study, we observed a successful elevation of serum zinc levels as well as significantly increased BMD at the lumbar spine, total hip, and femoral neck by oral zinc administration in both genders of elderly zinc deficient osteoporotic patients with a relatively higher responsiveness in women. In addition to standard therapy for osteoporosis, zinc supplementation may contribute to BMD augmentation ensuing the prevention of fracture occurrence in aged patients.

Regarding the involvement of zinc in bone formation, in vitro studies using osteoblastic MC3T3-E1 cells have demonstrated that zinc could promote cell proliferation and differentiation [18,19]. Zinc reportedly stimulates mRNA expression of runt-related transcription factor 2, which is a transcription factor related to differentiation of osteoblastic cells [20]. Furthermore, another important transcription factor involved in the osteoblast differentiation, osterix, is found to be a zinc finger transcription factor [21]. A significant augmentation of alkaline phosphatase activity, which is essential for bone mineralization, by zinc supplementation was shown in patients with traumatic bone fracture and in MC3T3-E1 cells [14,19]. Moreover, zinc has been demonstrated to upregulate the secretion of osteocalcin, as well as insulin-like growth factor-1 and transforming growth factor-beta1, from rat bone tissue with fracture healing and osteoblastic cells [13,22]. Therefore, zinc has been suggested to promote bone formation through the augmentation of bone growth factors and matrix proteins production, and the enhancement of osteoblastic cell proliferation, differentiation, and mineralization.

On the other hand, an inhibitory effect of zinc on bone resorption has also been demonstrated [23]. Zinc has shown to suppress osteoclastogenesis in mouse bone marrow cultures [24], and the effect is considered to be due to an inhibitory action on the receptor activator of nuclear factor-kappa B ligand (RANKL) system, which plays a pivotal role in osteoclast formation [25,26]. An enhanced expression of osteoprotegerin (OPG), a decoy receptor for RANKL, in osteoblastic cells by the zinc treatment was also reported [20,27]. Hence, zinc has thought to suppress osteoclastic activities via preventing RANKL/RANK/OPG signaling pathway. In sum, a trace element zinc is suggested to stimulate osteoblastic bone formation and mineralization, and inhibit osteoclastic bone resorption, thereby increasing bone mass.

In a clinical situation, several well-known osteoporosis-related risk factors are reportedly associated with decreased bone zinc amounts [8]. Significantly higher zinc excretion in urine was observed in women with postmenopausal osteoporosis [6,9], and there has been a reported association between low serum zinc levels and the risk of osteoporosis in postmenopausal women [10,11,12]. A beneficial effect of zinc treatment on fracture healing was demonstrated in patients with traumatic bone fracture as well as in an animal model [13,14]. In addition, a protective effect of oral zinc administration against bone resorption has also been suggested in rats [8].

The subjects of this study had consecutively received treatment for osteoporosis prior to and during the zinc pharmacotherapy. The medications included bisphosphonates, denosumab, teriparatide, and romosozumab. Bisphosphonates are the common drugs for treating osteoporosis which inhibit bone resorption by introducing osteoclasts into the apoptosis or cell death [28,29]. Denosumab is a fully human monoclonal antibody to RANKL that blocks the interaction with RANK, which thereby prevents differentiation and activation of osteoclastic cells [28,30]. On the other hand, teriparatide is a formulation of the N-terminal 34 amino acids of parathyroid hormone, and its intermittent administration activates the functions of osteoblasts rather than osteoclasts [28,31]. As a relatively new drug for osteoporosis, romosozumab, a humanized monoclonal antibody that binds and inhibits sclerostin, has been prescribed. Since sclerostin is a negative regulator of bone formation which inhibits Wnt signaling, romosozumab possesses a dual effect of increasing bone formation and decreasing bone resorption [32].

Although zinc administration was added-on to the standard treatment for osteoporosis in a clinical setting, BMD was significantly increased with augmented bone formation markers, P1NP and BAP. Moderately raised urinary NTx levels might be reflected by the enhanced bone turnover due to upregulated osteoblastic activities by zinc supplementation. Significantly positive associations between percentage changes of serum zinc and BMD suggest the possible efficacy of zinc pharmacotherapy for osteoporosis. This investigation contained 47 GIO patients (38.5%), of which 33 were suffering from RA. The results of the present study demonstrate an efficacy of zinc treatment for both primary and secondary osteoporosis with zinc deficiency.

As for the safety of zinc pharmacotherapy, nine patients of 131 participants experienced nausea and dropped out. A diminution of serum iron and copper has been concerned with zinc supplementation, however, no significant changes were observed in those parameters. In addition, no other serious adverse effects in physical conditions and/or laboratory data, as well as no incident fractures, were seen during the observation period.

This study had several limitations. First, the number of subjects was relatively small and limited to patients aged ≥65 years. Second, since zinc administration was added-on to the osteoporosis treatment in a standard practice, we could not precisely assess the effect of zinc supplementation. Future interventional trials on zinc administration without medication for osteoporosis, together with a larger sample size of participants, are required for further evaluation and understanding the efficacy of zinc supplementation for osteoporotic patients with zinc deficiency.

## 5. Conclusions

In conclusion, this investigation demonstrated a significantly increased BMD at the lumbar spine, total hip, and femoral neck, as well as a successful elevation of serum zinc levels, by oral zinc administration in elderly osteoporotic patients with zinc deficiency. Additive zinc supplementation to the osteoporosis treatment in a standard practice may contribute to BMD augmentation ensuing the prevention of fracture occurrence in aged patients. Further prolonged interventional trials with a larger sample size will be needed for understanding the precise effects of zinc treatment.

## Figures and Tables

**Table 1:** Baseline characteristics of study patients.

	Mean ± SD	Median (Minimum–Maximum)	Reference Value
Age, years	74.5 ± 7.1	75 (65–91)	
BMI, kg/m^2^	21.4 ± 3.3	21.8 (13.7–28.3)	18.5–25
Albumin, g/dL	4.1 ± 0.2	4.1 (3.6–4.4)	3.8–5.2
HbA1c, %	5.7 ± 0.4	5.8 (4.9–6.2)	4.6–6.2
eGFR, mL/min/1.73 m^2^	62.7 ± 10.8	63 (44.1–79.3)	≥60
Zinc, µg/dL	65.2 ± 9	63 (52–79)	80–130
Copper, µg/dL	114 ± 21	119 (73–143)	68–128
Iron, µg/dL	84.7 ± 19.2	82 (63–129)	48–200
Magnesium, mg/dL	2.1 ± 0.2	2.2 (1.7–2.4)	1.8–2.6
Calcium, mg/dL	9.3 ± 0.5	9.2 (8.7–10.7)	8.5–10.2
Phosphorus, mg/dL	3.6 ± 0.6	3.6 (2.1–4.7)	2.4–4.3
25(OH)D, ng/mL	19.2 ± 5	18 (12.8–29.6)	≥30
1,25(OH)_2_D_3_, pg/mL	56.5 ± 14.9	55 (32–85.7)	20–60
P1NP, ng/mL	27.7 ± 10.2	23.7 (18–55.9)	18.1–98.2
BAP, µg/L	9.8 ± 2.9	9.6 (5.8–16.1)	3.7–22.6
NTx, nmolBCE/mmolCre	37.1 ± 14.8	36.4 (14.4–57.5)	13–89
TRACP-5b, mU/dL	239 ± 83	233 (150–403)	120–590
Whole PTH, pg/mL	24.3 ± 8.2	23.3 (14.6–42.5)	8.3–38.7
Lumbar BMD, g/cm^2^	0.92 ± 0.21	0.84 (0.68–1.38)	1.15 ± 0.14 *
Total hip BMD, g/cm^2^	0.72 ± 0.12	0.72 (0.56–1.07)	0.96 ± 0.13 *
Femoral neck BMD, g/cm^2^	0.69 ± 0.12	0.66 (0.54–1.02)	0.94 ± 0.11 *
Osteoporosis treatment duration, years	4.7 ± 3.3	3.8 (1–11.5)	
Diabetes mellitus, yes	9 (7.4%)		
Dyslipidemia, yes	15 (12.3%)		
Hypertension, yes	34 (27.9%)		
Rheumatoid arthritis, yes	33 (27%)		
Prevalent osteoporotic fracture, yes	43 (35.2%)		

SD: Standard deviation; BMI: Body mass index; HbA1c: Hemoglobin A_1c_; eGFR: Estimated glomerular filtration rate; P1NP: Procollagen type 1 N-terminal propeptide; BAP: Bone-specific alkaline phosphatase; NTx: Cross-linked N-terminal telopeptide of type 1 collagen; TRACP-5b: Tartrate-resistant acid phosphatase-5b; PTH: Parathyroid hormone; BMD: Bone mineral density; * young adult mean ± standard deviation of Japanese women.

**Table 2:** Correlations between percentage changes of serum zinc and BMD.

	*r*-Value from Pearson’s Product-Moment Correlation	*P*-Value	*ρ*-Value from Spearman’s Rank Correlation	*P*-Value
vs. Lumbar BMD	0.477	<0.001	0.442	<0.001
vs. Total hip BMD	0.395	<0.001	0.321	<0.001
vs. Femoral neck BMD	0.452	<0.001	0.407	<0.001

BMD: Bone mineral density; vs.: Versus.

**Table 3:** Multiple regression analysis for the percentage changes of BMD by serum zinc percentage change (+1SD).

	Odds Ratio	95% CI	*P*-Value
for Lumbar BMD (+1SD)	1.81	1.39–2.37	<0.001
for Total hip BMD (+1SD)	1.77	1.37–2.23	<0.001
for Femoral neck BMD (+1SD)	1.86	1.42–2.42	<0.001

BMD: Bone mineral density; SD: Standard deviation; CI: Confidence interval. The regression models were adjusted for patient age, body mass index, and serum albumin and hemoglobin A_1c_ levels at registration.

## Data Availability

The datasets analyzed and/or generated during the current study are available from the corresponding author on a reasonable request.
